# What a pain in the … back: a review of current treatment options with a focus on naproxen sodium

**DOI:** 10.3389/jpps.2024.12384

**Published:** 2024-02-07

**Authors:** Steven M. Weisman, Giovanni Ciavarra, Grant Cooper

**Affiliations:** ^1^ Lumanity Clinical and Regulatory, Morristown, NJ, United States; ^2^ Princeton Spine and Joint Center, Princeton, NJ, United States

**Keywords:** opioids, low back pain (LBP), non-steroidal anti-inflammatory drugs (NSAIDs), naproxen, over-the-counter (OTC)

## Abstract

Non-specific low back pain (LBP) represents a challenging and prevalent condition that is one of the most common symptoms leading to primary care physician visits. While established guidelines recommend prioritizing non-pharmacological approaches as the primary course of action, pharmacological treatments are advised when non-pharmacological approaches are ineffective or based on patient preference. These guidelines recommend non-steroidal anti-inflammatory drugs (NSAIDs) or skeletal muscle relaxers (SMRs) as the first-line pharmacological options for acute or subacute LBP, while NSAIDs are the exclusive first-line pharmacological option for chronic LBP. Although SMRs are generally effective for acute LBP, the available evidence does not support the view that they improve functional recovery, and their comparative efficacy to NSAIDs and other analgesics remains unknown, while studies have shown them to introduce adverse events without significantly reducing LBP. Moreover, opioids continue to be widely prescribed for LBP, despite limited evidence for effectiveness and known risks of addiction and overdose. Broader use of non-opioid pharmacotherapy, including the appropriate use of OTC options, is critical to addressing the opioid crisis. The balance of evidence indicates that NSAIDs have a favorable benefit-risk profile when compared to other available pharmacological treatment options for non-specific LBP, a condition that is primarily acute in nature and well-suited for self-treatment with OTC analgesics. While clinical guidelines do not differentiate between NSAIDs, evidence indicates that OTC naproxen sodium effectively relieves pain across multiple types of pain models, and furthermore, the 14-h half-life of naproxen sodium allows sustained, all day pain relief with reduced patient pill burden as compared to shorter acting options. Choosing the most appropriate approach for managing LBP, including non-pharmacological options, should be based on the patient’s condition, severity of pain, potential risks, and individual patient preference and needs.

## Introduction

### Prevalence and classification

Low back pain (LBP) is one of the most common symptoms leading to primary care physician visits in the United States [1–3] and can be challenging to manage, since it is not a distinct disease entity and manifests with a diverse range of symptoms and severity. Healthcare providers play a pivotal role in guiding patients experiencing LBP toward effective treatment strategies.

An estimated up to 84% of adults will experience LBP [4], with a notable 25%–39% of U.S. adults reporting LBP episodes within the past 3 months [5, 6]. Ineffective management of LBP is associated with significant costs, since LBP is the leading cause of disability globally and ranks high among reasons for work absences and reduced productivity [7–11].

Acute LBP is usually considered non-specific, which means that it cannot be attributed to a specific disease or spinal pathology with certainty; it is often self-resolving [12], and individuals often manage their symptoms without seeking medical care [1, 13, 14]. LBP typically encompasses pain, stiffness, and/or discomfort in the lumbosacral region, commonly attributed to causes such as muscle sprains, ligament strains, herniated discs, osteoarthritis, scoliosis, traumatic injury, sciatica, and lumbar spinal stenosis. LBP may radiate to other parts of the body; individuals may experience symptoms such as muscle, hip, or leg pain, which can manifest as sharp, dull, aching, or burning sensations. The pain may vary in intensity and pattern, being intermittent, constant, or waxing/waning, and be accompanied by bilateral lower extremity paresthesia or exacerbated by movement [15–17].

LBP is classified according to its duration: acute (<4 weeks), subacute (4–12 weeks), and chronic (>12 weeks) [10, 16]. Acute LBP is the most common and often caused by mechanical issues or soft-tissue damage due to poor posture, prolonged sitting, improper lifting, muscle sprains, and injury or trauma. Patients with persistent symptoms often see continued improvement in the subacute phase; in primary care settings, an estimated 32% of patients transition from acute to chronic LBP [18]. This emphasizes the importance of effective treatment strategies during the acute and subacute periods to prevent the transition to chronic LBP, which accounts for much of the burden and costs of LBP.

### Treatment options

Established guidelines recommend non-pharmacological approaches as the first line of treatment, followed by pharmacological treatments only if non-pharmacological methods are ineffective or based on patient preference [10, 19–22]. Non-pharmacological approaches can include education on proper body mechanics, clinician-directed exercise programs (including formal physical therapy), local heat, massage/manipulation, acupuncture, electromyography biofeedback, low-level laser therapy, and cognitive behavior therapy [10].

Since many episodes of LBP are self-resolving, many individuals do not seek medical attention and may manage their symptoms over days to weeks using a combination of non-pharmacological and over-the-counter (OTC) pharmacological approaches. If these do not provide sufficient pain relief, they will often follow up with their healthcare professional. This provides an opportunity for healthcare professionals to assess and advise patients on appropriate pain relief options based on their distinct safety and efficacy profiles. This guidance can educate patients on proper dosing, precautions, and selection of the agent most suitable for their circumstances and needs. Both healthcare professionals and patients would benefit from an enhanced understanding of the benefits and risks of LBP treatment options, especially for those who are, or may be, at risk of adverse events or impacted by suboptimal treatment.

### Acute and subacute LBP

Guidelines published by the American College of Physicians (ACP) and endorsed by the American Academy of Family Physicians recommend non-steroidal anti-inflammatory drugs (NSAIDs) and skeletal muscle relaxers (SMRs) as first-line pharmacologic treatments for acute or subacute LBP [10, 23], as do the North American Spine Society and Department of Veterans Affairs [19, 20, 24]. The US Centers for Disease Control and Prevention (CDC) also support non-opioid medications, including NSAIDs, for LBP [25]. These guidelines do not distinguish between the various NSAIDs (e.g., naproxen, ibuprofen, and aspirin) [10, 19, 20, 24]. There are important differences between NSAIDs that can make one more suitable for an individual patient, as will be discussed later. Moreover, research across multiple types of pain models suggest that a single dose of naproxen is superior to other NSAIDs (ibuprofen and aspirin) and acetaminophen in terms of duration of pain relief [26–32], albeit there are studies suggesting that other NSAIDs may be superior to naproxen in some specific pain conditions [33, 34]. The occurrence of adverse events from OTC doses of naproxen is similar to that of placebo, with the most commonly reported mild-to-moderate adverse events related to the GI system [26–32]. In the context of managing LBP, naproxen alone demonstrated superior efficacy compared to combination therapies including: diazepam plus naproxen, cyclobenzaprine plus naproxen, oxycodone/acetaminophen plus naproxen, orphenadrine plus naproxen, or methocarbamol plus naproxen [35–38].

In a comprehensive review of 15 clinical practice guidelines containing recommendations for treating acute LBP, Oliveira et al. (2018) found a consensus highlighting NSAIDs as the primary choice for non-specific LBP [21]. Another analysis of clinical guidelines also found that they consistently recommend NSAIDs as the first-line pharmacological therapy [22], while international clinical practice guidelines provide conflicting recommendations for the use of SMRs [39].

Due to the lack of demonstration of meaningful effectiveness advantages with opioids in providing pain relief when compared to NSAIDs and the potential risks associated with long-term opioid use after treating acute pain, opioids are not recommended as first-line therapy for common acute pain conditions, including LBP [25, 40, 41], with one recent clinical study finding no significant difference in acute LBP severity between the opioid and placebo groups [42]. Opioids continue to serve an important role in managing pain related to severe traumatic injuries and major surgeries and in other instances when NSAIDs and other treatments are contraindicated or not likely to be effective.

### Chronic LBP

For patients with an inadequate response to non-pharmacologic therapy, NSAIDs are recommended as the exclusive first-line pharmacologic therapy, and SMRs are not recommended for chronic LBP [10]. Although guidelines for second-line pharmacologic therapy vary, they consistently recommend that opioids be a last resort, after a careful discussion of risks and realistic benefits with patients—when other treatments have failed, contraindication to NSAIDs exist, or the potential benefits outweigh the risks [10, 19, 20, 24].

In their analyses of clinical practice guidelines containing recommendations for the treatment of chronic LBP, investigators found a consensus highlighting NSAIDs as the primary choice for managing chronic non-specific LBP [21, 22].

## Pharmacological analgesics

The following sections provide an overview of the different pharmacological options for treating LBP, including acetaminophen, topical analgesics, NSAIDs, SMRs, opioids, antidepressants, systemic corticosteroids, and antiseizure medications. A focused discussion on the use of naproxen for LBP explores its efficacy evidence, safety profile, and potential benefits for managing LBP. The aim of the discussion is to provide a summary of available pharmacological options for LBP treatment, with an emphasis on assessing naproxen’s suitability based on its distinct pharmacological profile within the broader treatment landscape.

### Acetaminophen

While acetaminophen has an established role in pain management, clinical guidelines do not recommend it for treating LBP based on studies showing that it is ineffective at improving LBP outcomes versus placebo [10, 19, 20, 24, 43]. This lack of efficacy may be due to acetaminophen being a weaker analgesic than NSAIDs and with minimal anti-inflammatory activity, despite some overlapping mechanisms with NSAIDs [44, 45].

### Topical analgesics

Topical analgesic medications have a long history of use for managing a variety of acute and chronic pain conditions. Topical formulations provide localized delivery of active ingredients such as NSAIDs, anesthetics, and counterirritants such as menthol, methyl salicylate, and capsaicin. Studies indicate that topical NSAIDs, high concentration capsaicin, and lidocaine are effective for some pain conditions, with efficacy being highly dependent on the specific formulation and condition [46, 47]. By delivering high local drug concentrations at the site of pain while minimizing systemic absorption, they are optimal for pain confined to discrete areas like specific joints or skeletal muscles [48].

Since many non-specific LBP cases involve referred pain radiating from areas beyond the back itself, the effects of topical therapies directly applied to the low back can be limited. Currently, there is limited evidence supporting large or long-lasting analgesic effects from topical agents in treating LBP, especially relative to systemic pharmacological options like oral NSAIDs [49].

### NSAIDs

NSAIDs are well-established for pain management. Comprehensive reviews and clinical guidelines consistently support the view that, relative to placebo, NSAIDs reduce pain and improve function in patients with acute and chronic LBP; as a result, they are recommended as the first-line pharmacological therapy for LBP [10, 19, 20, 24, 49–51]. Moreover, studies have confirmed that NSAIDs, including naproxen, are as or more effective compared to other drugs alone or in combination with NSAIDs for the treatment of LBP [35–37, 52, 53].

Non-selective NSAIDs, such as naproxen and ibuprofen, work primarily by reversibly inhibiting the cyclooxygenase (COX) enzymes COX-1 and COX-2, which convert arachidonic acid into various compounds such as prostaglandins F2α (PGF2α) and E2 (PGE2) that are responsible for the pain and inflammation associated with many conditions [54–57]. While COX-1 is constitutively expressed and has key roles in the kidneys, gastrointestinal (GI) tract, and platelets, COX-2 expression is primarily induced during inflammation. This mechanism of action results in analgesic, anti-inflammatory, and antipyretic effects that make NSAIDs highly effective in treating a variety of pain conditions [54, 55].

While prescription NSAID regimens require healthcare provider supervision and per-patient assessment due to their risks, OTC equivalent regimens have an improved safety profile such that healthcare professional supervision is only required in some cases. The lower OTC doses and shorter treatment durations are associated with fewer side effects that are typically reversible upon discontinuation. However, as with any medication, risk cannot be entirely eliminated [58]. NSAIDs available as OTC formulations, such as naproxen sodium, have an established role in managing various types of pain, including LBP.

As mentioned previously, there are important distinctions between NSAIDs that can determine the suitability of one over another for an individual patient. Notably, studies indicate that a single dose of naproxen is superior to other NSAIDs (ibuprofen and aspirin) and acetaminophen in terms of duration of pain relief across several pain conditions [26–30, 32]. Moreover, naproxen has a pharmacokinetic profile that allows for a dosing interval of 8–12 h to achieve a sustained therapeutic effect. In contrast, other OTC NSAIDs must be readministered at 4–6-h intervals, which aligns with their maximum daily dosage and half-life. Naproxen’s extended dosing time frame reduces an individual’s overall “pill burden,” which has been identified as a factor linked to enhanced patient adherence [59] and enhances the consistency of serum concentrations, thereby delivering continuous pain relief while mitigating potential adverse effects tied to local exposure, peak concentrations, and subtherapeutic phases associated with fluctuations in pharmacokinetics [60]. It is noted that some epidemiological data suggest a slightly increased risk of GI bleeding with the use of low-dose naproxen compared with low-dose ibuprofen [61], which may possibly be attributed in part to its longer half-life [62], although a more recent analysis of controlled studies is not in agreement, finding no difference in adverse event profiles, including GI adverse events, between these regimens [31].

With longer duration of pain relief and comparable safety profile to other OTC NSAIDs, combined with an improved dosing regimen, naproxen is an ideal choice for OTC NSAID LBP management. The following sections provide additional details on naproxen in that context.

#### Naproxen background

Naproxen’s safety and efficacy has been established in clinical studies and its history of use. Naproxen and naproxen sodium (the salt form) belong to the arylpropionic acid class of NSAIDs [63]; naproxen sodium is more rapidly absorbed after oral administration [64] and thus provides a faster onset of action, making it more suitable for treating acute pain. Naproxen sodium achieves maximum concentration (Tmax) in 1-2 h (similar to the rate of absorption for ibuprofen), which is faster than naproxen (Tmax 2–4 h) [65, 66].

Oral naproxen sodium was approved in the United States first as a prescription drug, (Anaprox^®^) in 1974 and as an OTC analgesic (Aleve^®^) in 1994. In the United States, Aleve^®^ (≤660 mg/day) is indicated to temporarily relieve minor aches and pains associated with the common cold, headache, toothache, muscular aches, backache, arthritis, and menstrual cramps and to reduce fever in adults and children 12 years of age or older.

The U.S. OTC dosing of naproxen sodium is 220–440 mg as a single dose, every 8–12 h while symptoms persist, with a maximum total daily dose of 660 mg; however, in some countries, it is available with daily doses of 440–1,100 mg for OTC or prescription use. The approval of naproxen sodium for OTC administration was supported by more than 18 years of experience with prescription strength naproxen and naproxen sodium, and its efficacy and safety profile has been confirmed in a number of studies conducted before and after OTC approval.

#### Mechanism of action

Naproxen possesses the three properties now universally accepted as being characteristics of NSAIDs (analgesic, anti-inflammatory, and antipyretic effects), with its primary mechanism of action being the inhibition of the COX-2 enzyme, although it also inhibits COX-1 and is considered a non-selective NSAID [56]. Naproxen has been demonstrated to block the production of prostaglandins, which are responsible for the pain and inflammation associated with many conditions, via reversible and non-selective inhibition of COX-1 and COX-2 [55, 57], which support the production of prostaglandins F2α (PGF2α) and E2 (PGE2). Generally, prostaglandins generated from the COX-1 pathway elicit cytoprotective and homeostatic responses, whereas those from the COX-2 pathway elicit inflammatory responses [55].

#### Efficacy in managing LBP

Individuals with LBP need a variety of options to reduce or alleviate their pain. In many cases, naproxen represents an effective, long-lasting option based on its 14-h half-life, making all day pain relief a possibility. LBP is a common manifestation of pain originating from the muscles, and acute exacerbations can be managed effectively with OTC naproxen sodium, which is consistent with its current labeling as it is indicated for the temporary relief of minor aches and pains due to both backache and muscular ache.

In addition to the LBP studies described below, it is important to note that studies using muscle injury and post-dental-surgery pain models also provide high-quality evidence that is relevant to an assessment of naproxen’s efficacy in LBP. These additional models are discussed in the next section.

In one study, investigators evaluated the efficacy of naproxen (250 mg, three times daily for a total daily dose of 750 mg) compared to loxoprofen in non-surgical cases of LBP over a period of 6 weeks. Patients were evaluated at the end of the first, second, and sixth weeks. While there was no significant difference between the two treatment groups at any time point, both groups showed significant improvements over baseline, with the largest improvement observed after the first week of treatment [53]. Furthermore, two studies found that among patients with acute LBP, combining SMRs or oxycodone/acetaminophen to naproxen (500 mg; twice daily) did not improve outcomes compared to naproxen plus placebo [35, 36].

Although these studies use daily doses that are higher than the recommended OTC dose, the effects are expected to be similar, since naproxen has been shown to be effective across a range of pain models and dosage levels, including OTC doses. Overall, these findings indicate that naproxen, as a standalone treatment, is effective for managing LBP.

#### Leveraging the muscle injury and post-dental-surgery pain models as a bridge to assessing analgesic efficacy in LBP management

The efficacy of analgesic treatments is commonly established using randomized controlled trials conducted in patients with a defined pain condition and severity. Notably, muscle injury and post-dental-surgery pain provide an invaluable bridge for assessing the efficacy of analgesics in managing LBP, bolstering the body of evidence supporting the effectiveness of NSAIDs for this condition. Since NSAIDs have both analgesic and anti-inflammatory properties, it comes as no surprise that they are effective for relieving pain across various pain conditions, including muscle soreness and dental pain [26, 30, 32, 53, 67–74]. This is consistent with the current labeling of OTC naproxen sodium which is indicated for the temporary relief of minor aches and pains due to both toothache and muscular ache.

#### Muscle injury model

Muscle injury studies are applicable to LBP due to their focus on muscle soreness, which is a key contributor to LBP. Studies indicate that OTC doses of NSAIDs effectively reduce muscle injury, strength loss, and soreness [67, 70].

In a double-blind, crossover study, investigators examined the effects of naproxen sodium (220 mg, three times daily for a total daily dose of 660 mg) on exercised-induced muscle dysfunction, damage, and soreness. Participants were given either naproxen sodium or a placebo for 10 days after performing eccentric knee exercises. The study concluded that naproxen sodium attenuated muscle injury, strength loss, and soreness [67]. In another double-blind crossover study, the effectiveness of naproxen sodium (660 mg/day) versus placebo on muscle injury and soreness was assessed; it was superior to the placebo in improving muscle measurements and reducing thigh soreness through 4 days of recovery. This improvement was likely due to an attenuated inflammatory response to muscle injury [70].

These findings suggest that NSAIDs may provide similar relief for LBP by targeting a key source of pain.

#### Post-dental-surgery model

The post-dental-surgery pain model is a frequent and cost-effective approach to evaluate the efficacy of analgesics and may have higher assay sensitivity compared to other acute pain models [75–78]. This dental pain model provides a means to extrapolate analgesic efficacy to LBP since both conditions share a prostaglandin-driven nociceptive mechanism that is activated in response to tissue injury and inflammation (often due to trauma, disease, or chemical/thermal irritation) and triggers the sensation of pain [79–82].

The post-dental-surgery pain model requires only local anesthesia, allows for recruiting diverse participants with relative ease, and can be conducted under controlled conditions to minimize confounding factors affecting pain perception and response to interventions. Pain is induced in a standardized, reproducible manner enabling quantitative assessment using validated scales, enhancing the reliability and validity of results [75, 77, 78]. Furthermore, prescreening participants based on tooth extraction number and location helps predict postoperative pain levels. Overall, this pain model provides an efficient, controlled means of evaluating analgesic efficacy.

Multiple studies confirm that OTC doses of NSAIDs, including naproxen sodium and ibuprofen, effectively relieve post-surgical dental pain [26, 30, 68, 71]. In one study, the analgesic efficacy of naproxen sodium (440 mg), acetaminophen (1,000 mg), and placebo were compared in a single-dose, randomized, double-blind, 12-h study with patients with at least moderate pain secondary to extraction of three or four-third molars. Time to re-medication was significantly longer with naproxen sodium than with either acetaminophen or placebo. Moreover, naproxen sodium was also superior to acetaminophen for peak pain intensity difference, summed pain intensity differences, total pain relief, peak pain relief, time to reduction of pain by 50%, and overall rating [30]. Another study evaluated the efficacy of naproxen sodium (220 mg, three times daily for a total daily dose of 660 mg) as compared to a novel extended-release (ER) formulation of naproxen sodium over 24 h after extraction of one or two impacted third molars. It was reported that naproxen sodium (220 mg tid) and the novel ER formulation (660 mg) comparably and significantly reduced moderate to severe dental pain as compared to placebo. Significant pain relief was experienced from 15 min and sustained over 24 h, resulting in a reduced need for rescue medication [71]. Cooper et al. (2022) compared a single dose of naproxen sodium (440 mg) against hydrocodone plus acetaminophen (10/650 mg) in post-impaction surgery pain. For moderate-to-severe postsurgical dental pain, a single dose of naproxen sodium was at least as effective as hydrocodone plus acetaminophen in the early hours, significantly more effective at reducing pain intensity and providing greater pain relief over 12 h, and was better tolerated [68]. Lastly, Cooper et al. (2019) found that the duration of pain relief in subjects with moderate-to-severe post-surgical dental pain after a single dose of naproxen sodium (440 mg) was significantly longer than after a single dose of ibuprofen (400 mg). Furthermore, significantly fewer naproxen sodium-treated subjects required rescue medication over a 24 h period [26].

Additionally, the post-dental-surgery pain exhibited no notable differences in the estimate of analgesic efficacy when compared to other postsurgical pain models [83], and Dworkin et al. (2011) developed a comprehensive framework that outlines the level of efficacy evidence necessary to extrapolate specific model findings to other types of pain [84]. Specifically, evidence of efficacy in three types of acute pain (postoperative pain; pain associated with non-surgical trauma; and disease-associated visceral pain) was established as the basis for extrapolation to other acute pain conditions. Evidence of efficacy in two different acute pain conditions is a well-established and accepted pathway for testing analgesic efficacy of a general pain reliever and is used to extrapolate to a general pain indication. Naproxen meets these criteria, providing additional support for the extrapolation of efficacy data across various pain models [27, 32, 70, 71, 85–87].

#### Safety profile

At OTC doses and durations, naproxen is typically well tolerated and safe [58], but its mechanism of action, which is shared with other NSAIDs, has been linked to GI, CV, and renal adverse effects. Several advisory committees recognized that naproxen, similar to all other NSAIDs, is associated with a small, yet noteworthy, elevated CV risk which is lower compared to ibuprofen at higher doses [88, 89]. However, no apparent differences are observed at OTC doses [31]. Nevertheless, the FDA issued a label mandate requiring all OTC NSAIDs to advise consumers to ask their doctor if they have a history of stomach problems, CV disease, or kidney disease. In specific underlying conditions or situations with an increased risk of bleeding, patients are advised to seek medical advice: advanced age, ulcers, bleeding problems, use of prescription NSAIDs, excessive alcohol consumption, or taking the product longer than directed [88, 90].

A comprehensive analysis of naproxen’s clinical pharmacology and CV safety highlighted that the low COX-2 selectivity of naproxen results in a lower CV risk compared to other NSAIDs, as CV risk is associated with COX-2 selectivity. Consequently, the authors concluded that “*the over-the- counter use of naproxen is expected to pose minimal cardiovascular risk*” [88]. Moreover, another review concluded that “*Current evidence suggests that naproxen, a non*-*selective NSAID, is associated with the lowest risk of cardiovascular events. Therefore, naproxen is the NSAID of choice in patients with high cardiovascular risk*” [91]. Notably, joint recommendations by several medical societies, including the Asian Pacific Association of Gastroenterology, Asia Pacific League of Associations for Rheumatology, and Asia-Pacific Society for Digestive Endoscopy, now include naproxen as one of the preferred drugs for patients with high CV risk if NSAID use is unavoidable [92]. A recent observational study analyzing NSAID prescription claims post-myocardial infarction confirmed the association between increased CV and bleeding risks from NSAID use in this population. Among the NSAIDs, celecoxib and meloxicam exhibited the least increase in adverse outcomes, and therefore, it has been suggested that if NSAID use is medically necessary in this patient population, celecoxib or meloxicam may represent viable options [93, 94]. In addition, while there are reports suggesting that concomitant use of some non-aspirin NSAIDs interfere with the aspirin’s anti-platelet function and possibly reducing its CV benefits [95], there are data showing that aspirin retains its cardioprotective effects in the presence of naproxen, ibuprofen, meloxicam, and rofecoxib [96].

Observational studies indicate a higher risk of CV and renal events with higher NSAID doses and duration, while showing a significant decrease in CV events when comparing OTC doses and durations relative to prescription regimens [97]. For example, one study found that prescription naproxen use was not associated with an increased risk of major vascular events [90]. While these epidemiological data certainly support the CV and renal safety of OTC naproxen, these data must be interpreted with caution since they are are susceptible to confounding factors, like all observational studies. Recently, the PRECISION randomized controlled trial concluded that prescription doses of the three agents compared (naproxen, celecoxib, and ibuprofen), were associated with similar risks of major adverse cardiac events [89].

It is well established that COX-1 inhibition can lead to GI adverse effects, and OTC naproxen is associated with elevations in mild effects (constipation, diarrhea, dyspepsia, and nausea) but, in contrast with prescription dosages, the elevation is not significantly or clinically different. In a pooled analysis of naproxen studies with OTC dosages, GI adverse events were non-significantly elevated with naproxen versus placebo [98]. Similar to CV risk, evidence suggests that the risk of GI complications is minimized at OTC dosages and durations.

All NSAIDs can impair kidney function by inhibiting COX-1 and COX-2 in the kidneys [99]. It is thought that the increased CV risk among NSAID users stems from elevated blood pressure caused by COX-2 inhibition in the kidneys [88]. However, this effect is not seen at OTC doses, and since naproxen does not significantly raise systolic blood pressure, this may contribute to its more favorable safety profile compared to other NSAIDs [88].

Safety concerns related to naproxen primarily involve GI, CV, or renal risks when taken at prescription doses and for prolonged periods. However, at OTC dosages, the risk is lower as compared to prescription doses, and elevated risks identified in large cohort studies often did not reach statistical significance, including in participants with higher baseline risks. When assessing the suitability of naproxen for individuals with LBP, it is important to evaluate the balance between its benefits and risks on a case-by-case basis taking into account any preexisting conditions that might elevate risks for adverse events. Moreover, since naproxen is non-addictive, it offers a means for healthcare providers and individuals to steer clear of the effects linked to opioid dependency discussed in more detail in the following section.

### Opioids

Guidelines recommend against opioids as first-line pharmacological therapy, yet these continue to be prescribed for LBP despite their unfavorable safety profile and general lack of evidence to support their effectiveness [40, 100–102]. Moreover, published studies have demonstrated that NSAIDs are as or more effective compared to opioids or opioids combined with NSAIDs for relief of LBP, despite the latter being associated with addiction and overdose-related mortality, a risk that has increased alongside prescription rates [40]. In one study, adding oxycodone/acetaminophen to naproxen 500 mg twice daily found no improvement in efficacy (pain relief and functional improvement) over naproxen alone for acute LBP prompting the authors to conclude that “*These findings do not support use of these additional medications [beyond naproxen] in this setting*” [35]. Moreover, the SPACE RCT compared opioids against non-opioids (including NSAIDs) for improving pain-related function in subjects with hip/knee osteoarthritis and chronic back pain. Over a 12 months period, opioids did not exhibit superior improvements in pain-related function compared to non-opioids [52].

Opioids are not recommended as first-line treatment choice for many common acute pain conditions because of the potential risks associated with their long-term use post-treatment. Opioids for acute pain can inadvertently lead to prolonged usage if prescribers provide large supplies or prescriptions are continuously refilled, resulting in drug dependence [40, 103]. Limited evidence supports improved pain or function with long-term use of opioids for several chronic pain conditions for which they are commonly prescribed, including chronic non-specific LBP. In some cases, evidence indicates worse outcomes associated with prolonged opioid usage for these conditions [10, 104]. Moreover, studies that have assessed opioids for chronic LBP have not addressed the risk for addiction, abuse, or overdose, although data show a dose-dependent relationship between opioid use for chronic pain and serious harms [105]. Furthermore, an analysis of patients who visited an emergency department with LBP showed that they were significantly more likely to return within 6–12 months with LBP complaints if they were prescribed an opioid at discharge compared to patients who were not. Receiving opioids at discharge also doubled the odds of return within 12 months, while receiving NSAIDs reduced the odds by 60% [106].

Data from the CDC indicate that in 2021 there were 16,706 reported deaths involving prescription opioids; almost 5 times higher than in 1999 [107]. The use of opioids carries many possible adverse effects, some of which are serious and life-threatening. GI effects like constipation, nausea, and vomiting are well-known risks associated with long-term opioid use [108, 109]. Additional but less frequent adverse effects include cardiovascular depression (bradycardia, hypotension), headaches, hypothermia, inability to urinate, muscle and bladder spasms, muscle rigidity, flushing, and involuntary muscle twitching and/or jerking. Opioid-induced hyperalgesia, an effect that heightens rather than dulls pain, has also been reported as a potential adverse effect [110, 111].

To reduce dependence on opioids, it is important that healthcare providers prioritize non-opioid treatment approaches, explore comprehensive pain management strategies, and adhere to established clinical guidelines, which generally recommend non-pharmacological approaches as the initial treatment for LBP, followed by NSAIDs as the first-line pharmacological therapy. Opioids should be considered the last resort due to the uncertain efficacy and risks associated with addiction and overdose. It is important to establish an adequate treatment plan concordant with established guidelines to reduce the risk of opioid dependence or transitioning from acute/subacute pain to chronic pain.

### Skeletal muscle relaxers

Centrally acting SMRs are approved for acute musculoskeletal conditions, and their prescribing doubled between 2005 and 2016 [112]. They are the third most commonly prescribed drug for LBP [39]. Although they are generally effective for acute LBP, the body of evidence originates from specific SMRs, their comparative efficacy to NSAIDs and other analgesics remains unknown, and evidence is lacking to support their use for chronic LBP [39, 113, 114]. Furthermore, it was shown that combining SMRs (i.e., orphenadrine or methocarbamol) to 500 mg by mouth naproxen (twice daily) for acute LBP did not increase efficacy (pain relief and functional improvement) when compared to naproxen plus placebo [36].

Based on moderate-quality evidence, the ACP recommends NSAIDs or SMRs as first-line pharmacological treatment for acute LBP [10], although the evidence does not support the view that SMRs improve functional recovery [115]. Studies have shown that SMRs introduce adverse events without significantly reducing LBP [39, 116]. SMRs have well-established central nervous system adverse effects, such as drowsiness and dizziness, and, due to limited long-term efficacy and safety data coupled with the potential risk for abuse, dependence, and overdose, their use is generally recommended for a maximum of 2-3 weeks [117–119]. Despite these limitations, one estimate suggests that 18.5% of LBP patients receive an SMR (unfortunately, the investigators were not able to distinguish between acute and chronic LBP) [120] and SMR use was found to increase rapidly between 2005 and 2016 [112]. This trend that may have taken hold as healthcare providers and patients seek alternatives to opioids for the management of LBP and other conditions. Overall, it is important to exercise caution when recommending SMRs for LBP, since the evidence suggests they may only be effective for acute and subacute LBP, and caution is especially warranted for patients with comorbidities, underlying conditions, or a history of substance abuse [112, 121].

### Antidepressants, systemic corticosteroids, and antiseizure medications

Although the data supporting antidepressants’ efficacy remains uncertain, estimates indicate that roughly 25% of U.S. primary care physicians prescribe them for LBP [122]. The rationale is that individuals with chronic LBP often exhibit concurrent depression, and addressing the depression can raise pain tolerance. Some antidepressants are believed to have analgesic mechanisms distinct from their mood-elevating ones, and their sedative effects are thought to ameliorate insomnia in patients with chronic LBP. Nevertheless, the evidence does not generally support antidepressants for LBP treatment, as several studies have observed only a marginal and clinically non-significant benefit, primarily with SNRIs [21, 122–125]. Furthermore, antidepressants are associated with a variety of side effects, including dry mouth, constipation, drowsiness, dizziness, weight gain, and sexual issues, which collectively are a common reason for patients’ discontinuation of them [126, 127].

Corticosteroids possess both anti-inflammatory and immunosuppressant properties, and their side effect profiles can be extensive and affect many organ systems [128]. In general, no differences in efficacy for pain have been reported between systemic corticosteroids and placebo; a small effect on function in patients with radicular LBP has been observed, but the effects in individuals with non-radicular LBP remain uncertain [10, 129, 130]. Before initiating corticosteroids for LBP, healthcare providers should consider their potential adverse effects and patients’ underlying comorbidities to ensure any potential benefits outweigh the harms.

Antiseizure medications are commonly prescribed to treat LBP despite inconclusive evidence for the treatment of LBP [10, 21, 123, 124, 131] despite their increased risk of adverse events, including suicidality [132–134].

## Discussion

The balance of evidence indicates that NSAIDs, including naproxen, have a favorable benefit-risk profile when compared to other available pharmacological treatment options for non-specific LBP, a condition that is primarily acute in nature and well-suited for self-treatment with OTC analgesics. While there are a variety of prescription analgesic options, the evidence supporting their effectiveness is generally limited, and they carry risks of significant adverse effects. SMRs appear to be effective for acute LBP, but their risk for dependence and central nervous system side effects must be carefully considered.

Managing acute LBP with NSAIDs is supported by several clinical guideline recommendations, which endorse exhausting non-opioid options before considering opioids. Broader use of non-opioid pharmacotherapy, including the appropriate use of OTC options, is critical to addressing the opioid crisis.

While the recommendations do not differentiate between NSAIDs, evidence indicates that OTC naproxen sodium effectively relieves pain comparable to a common opioid in a dental pain model. Naproxen sodium’s 14-h half-life allows sustained, all day pain relief with reduced patient pill burden as compared to shorter acting options.

Choosing the most appropriate approach for managing LBP, including non-pharmacological options, should be based on the patient’s condition, severity of pain, potential risks, and individual patient preference and needs. In conclusion, naproxen sodium offers an effective non-opioid pharmacological option for LBP with advantages compared to other OTC NSAIDs.
